# An Atlas of the Normal and Pathological Nervous Systems

**Published:** 1896-07

**Authors:** 


An Atlas of the Normal and Pathological Nervous Systems, together with a Sketch of the Anatomy, Pathology and Therapy of the same. By Dr. Christfried Jakob, practising physician in Bamberg ; formerly First Assistant in the Medical Clinic at Erlangen. With an introduction by Prof. Dr. Ad. v. Strümpell. Translated and edited by Joseph Collins, M. D., Instructor of Mental and Nervous Diseases at the New York Post-Graduate Medical School. New York : William Wood & Co. 1896.

The publishers are to be congratulated for securing as translator of this work so able and competent a man as Dr. Joseph Collins, whose knowledge of the subject and of the language enable him to add materially to the worth of the volume.

It is divided into five parts, part one treating of the morphology of the central nervous system ; part two, of the development and structure of the nervous system ; part three, of the topographical anatomy of the nervous system ; part four, of the general pathological anatomy of the nervous system ; and part five, of the special pathology of the spinal cord and the peripheral nerves. Seventy-eight full-page plates, many containing from one to five figures, besides numerous figures in the text, constitute the working basis, the text serving to elucidate the plates rather than the plates to elucidate the text, as is generally the case.

The figures are nicely executed, many in colors, while some are so arranged as to show different layers or different depths of the brain.

If the other volumes of the series are as valuable and attractive as this one, the publishers may expect hearty appreciation from the profession for their labors and efforts. W. C. K.

